# Accuracy of Diagnostic Methods and Surveillance Sensitivity for Human Enterovirus, South Korea, 1999–2011

**DOI:** 10.3201/eid.1908.130496

**Published:** 2013-08

**Authors:** Ji-Yeon Hyeon, Seoyeon Hwang, Hyejin Kim, Jaehyoung Song, Jeongbae Ahn, Byunghak Kang, Kisoon Kim, Wooyoung Choi, Jae Keun Chung, Cheon-Hyun Kim, Kyungsoon Cho, Youngmee Jee, Jonghyun Kim, Kisang Kim, Sun-Hee Kim, Min-Ji Kim, Doo-Sung Cheon

**Affiliations:** Korea Center for Disease Control and Prevention, Cheongwon-gun, Chungcheongbuk-do, South Korea (J.-Y. Hyeon, S. Hwang, H. Kim, J. Song, J. Ahn, B. Kang, Kisoon Kim, W. Choi, Kisang Kim, D.-S. Cheon);; Public Health and Environment Institute of Gwangju, Gwangju, South Korea (J.K. Chung, S.-H. Kim, M.-J. Kim);; Public Health and Environment Institute of Jeollabukdo, Imsil-gun, Jeollabukdo, South Korea (C.-H. Kim);; Public Health and Environment Institute of Busan, Busan, South Korea (K. Cho);; World Health Organization, Western Pacific Region, Manila, Philippines (Y. Jee);; Catholic University College of Medicine, Suwon, Kyeonggido, South Korea (J. Kim)

**Keywords:** human enterovirus, surveillance, prevalence, genotype, cell culture, RT-PCR, real time RT-PCR, the Republic of Korea, South Korea, Korea Centers for Disease Control and Prevention, viruses

## Abstract

The epidemiology of enteroviral infection in South Korea during 1999–2011 chronicles nationwide outbreaks and changing detection and subtyping methods used over the 13-year period. Of 14,657 patients whose samples were tested, 4,762 (32.5%) samples were positive for human enterovirus (human EV); as diagnostic methods improved, the rate of positive results increased. A seasonal trend of outbreaks was documented. Genotypes enterovirus 71, echovirus 30, coxsackievirus B5, enterovirus 6, and coxsackievirus B2 were the most common genotypes identified. Accurate test results correlated clinical syndromes to enterovirus genotypes: aseptic meningitis to echovirus 30, enterovirus 6, and coxsackievirus B5; hand, foot and mouth disease to coxsackievirus A16; and hand, foot and mouth disease with neurologic complications to enterovirus 71. There are currently no treatments specific to human EV infections; surveillance of enterovirus infections such as this study provides may assist with evaluating the need to research and develop treatments for infections caused by virulent human EV genotypes.

Human enteroviruses (EVs) belong to the family *Picornaviridae*, genus *Enterovirus*, and are classified into 4 species, EV-A, B, C, and D (*1**–**3*). More than 90 serotypes are currently recognized by the International Committee on Taxonomy of Virus Classifications. EV-A (17 serotypes), EV-B (56 serotypes), EV-C (16 serotypes), and EV–D (3 serotypes) species classifications are based on similarities in virus capsid protein (VP) genes (*4**–**6*). Among them, 65 serotypes are known to cause infections in humans, including polioviruses, echoviruses (E), coxsackieviruses A (CA) and B (CB), and EV types 68–71 (*7**,**8*).

Most EV infections (hand, foot and mouth disease [HFMD]; gastroenteritis; and acute hemorrhagic conjunctivitis) are asymptomatic or mild, and infected persons can recover without specific medication (*5**,**8**–**10*). However, the neurotropism of some EVs can cause serious central nervous system complications such as aseptic meningitis, encephalitis, and flaccid paralysis (*9**,**11**, **12*). Although some EVs cause severe and potentially life-threatening illness, there is currently no antiviral treatment available for EV infection (*9*).

Laboratory diagnosis of EV infection is based on detection of the virus in clinical specimens such as fecal or rectal swab samples, cerebrospinal fluid (CSF), nasopharyngeal secretions collected by throat swab, and blood (*11**,**13*). Detection of EV is usually performed by isolation of the virus in cell culture, reverse transcription PCR (RT-PCR), or real-time RT-PCR (*11**,**14**–**16*). Currently, RT-PCR is used routinely worldwide to diagnose EV infection because of its sensitivity, specificity, and ability to detect highly conserved 5′ noncoding regions of the human EV genome (*15**,**17**,**18*). For determining subtype, the neutralization test is the standard diagnostic tool and is generally reliable, but it is also labor-intensive, time-consuming, and may fail to identify an isolate (*16**,**19*). Therefore, RT-PCR amplification of the VP1 coding region, then amplicon sequencing, is a sufficient mechanism for molecular typing of EVs (*20*).

Since 1993, the national enterovirus surveillance system of the Korea Centers for Disease Control and Prevention (KCDC) has monitored and characterized human EV infection in patients with EV-related diseases. Three basic detection methods for diagnosis have been used in this system since surveillance began. During 1993–2004 (phase I), cell culture methods were used; during 2005–2007, RT-PCR was used (phase II); and from 2008–2011 (phase III), real-time RT-PCR was the standard detection method used. Before 2005, genotyping was performed by using the neutralization test, but since then, as documented for phases II and III of this study, VP1 sequencing was used to genotype EV. In this study, we obtained the clinical and epidemiologic data regarding enterovirus infections, including outbreaks and sporadic cases, during 1999–2011 in South Korea, and focused on the improvement of surveillance sensitivity as diagnostic methods developed.

## Materials and Methods

### Surveillance System and Data Sources

The KCDC national enterovirus surveillance system consists of 180 clinics managed by pediatrics physicians (35 primary clinics, 105 secondary hospitals, and 40 tertiary hospitals nationwide), and the number of clinics participating in the surveillance system varied each year. Participating physicians collected specimens from patients whose illnesses included meningitis, encephalitis, influenza-like illness, HFMD, herpangina, and gastroenteritis, and documented patient age, date of specimen collection, symptoms, and suspected diagnosis. KCDC registered information on a website (http://enterovirus.macrogen.com/cdclab/) originally set up in 2009. Analysis of the specimens, including typing of relevant EVs and other characterizations, was done at the National Polio Laboratory of KCDC.

### Patients and Samples

In total, 17,349 clinical samples from 14,657 patients with suspected enteroviral disease were collected during January 1999–December 2011. The average ages of the patients from primary clinics, secondary hospitals, and tertiary hospitals were 13, 5, and 8 years, respectively. Sample types investigated were as follows: 9,012 fecal samples; 5,045 CSF samples; 1,979 throat swab samples; 516 blood samples; and 804 other samples, including urine, saliva, pericardial fluid, and skin swab. Fecal samples are the most common samples obtained from patients suspected of having enterovirus infections. CSF samples were collected from the patients in secondary and tertiary hospitals who had meningitis or encephalitis, and throat swab samples were collected from those with influenza-like illness. 

### EV Detection in Clinical Samples

Over 13 years, 3 methods were used to detect EV in South Korea. We identified the periods during which the methods were used as phases I, II, and III.

#### Phase I (1999–2004): Cell Culture

During 1999–2004, clinical samples were processed by using the World Health Organization (WHO) polio laboratory manual as follows (*21*). Fecal material was made into a suspension (10%) by dilution with 0.1mol/L phosphate-buffered saline, and 10% (vol/vol) chloroform was added. The mixture was then vigorously shaken for 5 min and centrifuged at 500 × *g* for 15 min. Following centrifugation, the supernatant was transferred to a new tube and then injected into cells. Pharyngeal swab samples were collected in virus transport medium; CSF, serum, and pericardial fluids were directly injected into cells. Rhabdomyosarcoma and L20B cell lines were used to isolate the EVs.

#### Phase II (2005–2007): RT-PCR

During 2005–2007, viral RNA was extracted from each sample by using magnetic beads (GM-Autoprep Kit, Seoul, South Korea) according to the manufacturer’s instructions, and the purified viral nucleic acid was processed by using Freedom EVO (Tecan, Männedorf, Switzerland). RT-PCR was performed by using primers designed in a previous study (*14*).

#### Phase III (2008–2011): Real-time RT-PCR

During 2008–2011, one-step real-time RT-PCR was performed by using a dually labeled fluorogenic EV-specific probe and primers. A highly conserved 5′ noncoding region was the target of a previously described 196-bp region (*15**,**22*).

### Characterization of EV

#### Phase I: Neutralization Test 

Cell culture isolates were identified by using neutralization tests consisting of standard polyclonal antiserum typing according to WHO recommendations (*21**,**23*). Two reference-typing serum sources were used for microneutralization tests: the Lim-Benyesh–Melnick equine antiserum pools supplied by the WHO Collaborating Centre for Virus Reference and Research and the RIVM pools (National Institute of Public Health and the Environment, Bilthoven, the Netherlands).

#### Phases II and III: Partial Sequencing of the VP1 Genomic Region

For genotyping, the VP1 amplicon (375 bp) was amplified by seminested RT-PCR and then sequenced according to the US CDC protocol (*24*). To determine the EV genotype, we compared the sequence homology between the amplified PCR products and the VP1 sequences available from GenBank. The sequences obtained were identified in terms of closest homology by using BLAST (http://blast.ncbi.nlm.nih.gov/Blast.cgi).

## Results

### Prevalence of EV and Improvement of Surveillance Sensitivity

**Figure 1:**
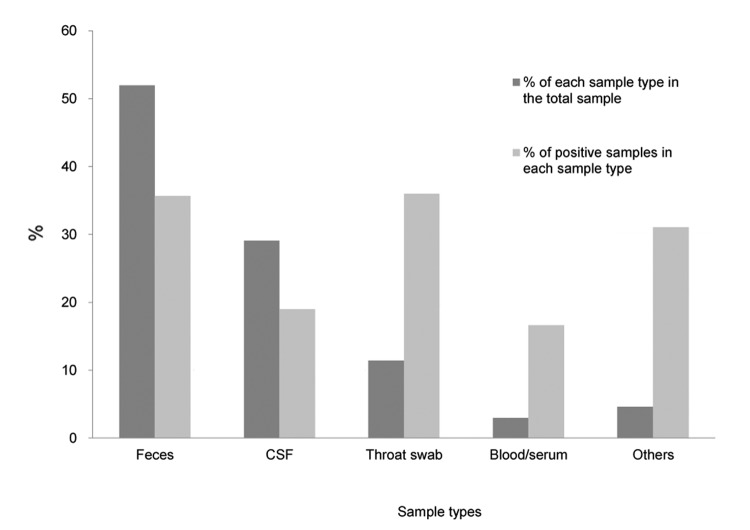
Specimens submitted for detection of enterovirus (n = 17,349) and proportions with positive results. Other samples included urine, saliva, pericardial fluid, and skin swab.

Of 17,349 specimens collected during 1999–2011, a total of 5,220 (30.1%) were laboratory confirmed as EV positive (Figure 1). Fecal or rectal swab samples, the most commonly collected sample type, accounted for 9,012 (51.9%) of all samples collected, and 3,213 (35.7%) of those samples were positive for EV (Figure 1). For other sample types, 19.0% (958/5,045) of CSF, 36.0% (713/1,979) of throat swab samples or secretions, 16.7% (86/515) of blood, and 31.3% (250/798) of other samples (i.e., urine, saliva, pericardial fluid, and skin swabs) were confirmed positive for EV.

The annual prevalence of EV during 1999–2011 is shown in Table 1. The EV detection rate varied each year, ranging from 3.8% in 2001 to 54.2% in 2008. A total of 4,762 (32.5%) of 14,657 patients were infected during 1999–2011. By use of the cell culture method during phase I (1999–2004), 20.5% were detected; by use of RT-PCR during phase II (2005–2007), 26.4% were detected; and by use of real-time RT-PCR during phase III, (2008–2011), 39.2% were identified.

**Table 1:** Analysis of diagnostic methods for detecting human EV and surveillance outcomes, South Korea, 1999–2011*

Year	No. samples	No. (%) positive	Average % positive
Phase I†			20.5
1999	372	133 (35.8)	NA
2000	261	30 (11.5)	NA
2001	676	26 (3.80)	NA
2002	1,272	361 (28.4)	NA
2003	264	66 (25.0)	NA
2004	314	33 (10.5)	NA
Phase II‡			26.4
2005	890	382 (42.9)	NA
2006	1,059	238 (22.5)	NA
2007	1,131	193 (17.1)	NA
Phase III§			39.2
2008	2,332	1,264 (54.2)	NA
2009	2,766	869 (31.4)	NA
2010	1,477	566 (38.3)	NA
2011	1,843	601 (32.6)	NA
Total	14,657	4,762 (32.5)	NA

*EV, enterovirus; NA, not applicable. †During phase I, human EV was detected and serotyped by using cell culture. ‡During phase II, human EV was detected by reverse transcription PCR (RT-PCR) and genotyped by sequencing of virus capsid protein (VP) 1 region. §During phase III, human EV was detected by real-time RT-PCR and genotyped by sequencing of the VP1 region.

### Distribution of Enterovirus Infection by Season and Age

**Figure 2:**
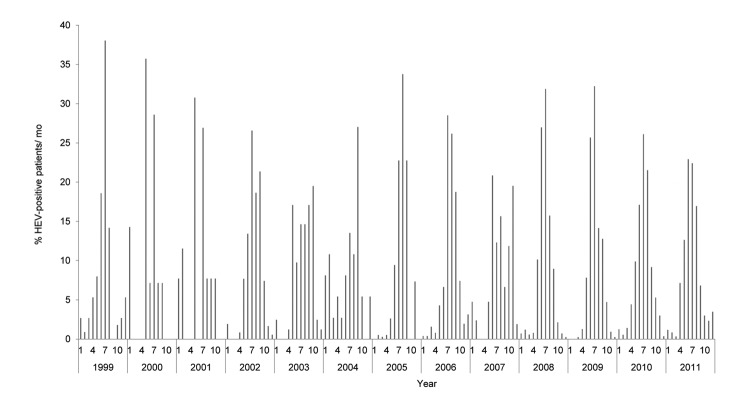
Seasonal pattern of enterovirus circulation during 1999–2011. Bars indicate percentage of patients positive for human enterovirus per month.

The EV detection rate varied throughout each year (Figure 2); the number of EV cases increased during late spring, summer, and the beginning of autumn (May–September) (Figure 2). The peak months of detection were as follows: July in 1999, May in 2000 and 2001, July in 2002, October in 2003, September in 2004, August in 2005, July in 2006, June in 2007, July in 2008–2010, and June in 2011 (Figure 2). Low detection rates (<10%) were generally observed during late autumn into early spring (October–April) except during January in 2000, February in 2001 and 2004, October in 2003 and 2007, and November in 2007 (Figure 2).

Age was known for 12,296 of the 14,657 patients studied. Age distribution of the 4,209 patients whose ages were known and test results were positive, as shown in Table 2, was 980 (23. 3%) patients <1 year of age, 1,846 (43.9%) 2–5 years of age, 937 (22.3%) 6–10 years of age, 285 (6.8%) 11–20 years of age, 60 (1.4%) 21–30 years of age, 68 (1.6%) 31–40 years of age, 17(0.4%) 41–50 years of age, 13 (0.3%) 51–60 years of age, and 3 (0.07%) >60 years of age.

**Table 2:** Age distribution of human enterovius patients, South Korea, 1999–2011

Age, y*	No. positive/total persons in age group	Total (%)

1999	2000	2001	2002	2003	2004	2005	2006	2007	2008	2009	2010	2011
0–1	23/48	5/51	1/131	30/145	7/35	12/315	130/252	73/318	52/336	260/624	10/40	145/579	232/877	980 (23.3)
2–5	36/86	3/33	9/90	143/369	27/44	8/46	113/202	65/189	78/230	309/529	529/1,628	288/470	238/429	1,846 (43.9)
6–10	26/76	2/34	6/71	73/240	5/20	8/70	63/204	31/96	1/75	300/446	264/619	82/172	76/174	937 (22.3)
11–20	13/38	1/19	1/49	48/162	2/26	0/6	12/64	10/60	1/26	70/158	63/425	29/150	35/168	285 (6.8)
21–30	2/7	0/3	1/11	9/25	0/2	0/10	2/11	6/13	0/8	31/69	0/14	4/21	5/91	60 (1.4)
31–40	1/6	0/2	0/3	5/13	6/8	0/4	3/10	1/11	0/11	39/58	3/16	8/30	2/56	68 (1.6)
41–50	2/9	0/3	0/3	6/16	4/6	0/3	2/8	0/10	0/1	0/12	0/11	0/9	3/15	17 (0.4)
51–60	0/4	0/3	0/3	4/6	2/3	2/3	0/6	1/7	0/1	1/7	0/7	1/9	2/2	13 (0.3)
>60	0/5	0/1	0/3	3/6	0/2	0/2	0/4	0/8	0/3	0/8	0/5	0/5	0/0	3 (0.1)
Total	103/279	11/149	18/364	321/982	53/146	30/279	325/761	187/712	132/691	1,010/1,911	869/2,765	557/1,445	593/1,812	4,209

*Age known for 12,296 persons.

### Clinical Manifestations and Genotypes of EV

During the period studied, 44 different genotypes were detected among 3,128 EV-positive samples (Table 3). The 5 main genotypes were enterovirus (EV) 71, echovirus (E) 30, coxsackieviruses B (CB) 5, E6, and CB2, accounting for 14.9%, 12.5%, 9.3%, 8.4%, and 6.0% of the total EV, respectively. The 5 most frequently observed genotypes during each phase were phase I: E6, E13, E9, polio Sabin strain, and CB2; phase II: CB5, E18, CB3, CB2, and E25; and phase III: EV71, E30, E6, CA16, and CB5. In addition, 39 polioviruses had been detected before 2006 and confirmed as being related to the polio Sabin strain (data not shown).

**Table 3:** Five most frequent human enterovirus genotype rankings during the epidemic seasons in South Korea, by year, 1999–2011*

Year	Genotype, no. (%)				
	Rank 1	Rank 2	Rank 3	Rank 4	Rank 5
Phase 1†					
1999, n = 85	CB2, 21 (24.7)	E6, 15 (17.7)	CB3, 10 (11.7)	E11, 8 (9.4)	E30, (8 (9.4)
2000, n = 30	EV71, 12 (40)	Polio Sabin strain, 9 (30)	CB2, 2 (2.7)	E6, 2 (2.7)	E11, 2 (2.7)
2001, n = 26	CB5, 12 (46.2)	CB3, 4 (15.4)	Polio Sabin strain, 3 (11.6)	CB2, 3 (11.6)	CB1, 3 (11.6)
2002, n = 272	E13, 70 (25.7)	E9, 59 (21.7)	E6, 53 (19.5)	E7, 24 (8.8)	CB3, 17 (6.3)
2003, n = 54	CB4, 16 (19.5)	E6, 10 (12.2)	E30, 7 (8.5)	CB1, 7 (8.5)	Polio Sabin strain, 5 (6.1)
2004, n = 29	Polio Sabin strain, 7 (24.1)	E30, 6 (20.7)	CB2, 5 (17.3)	CB1, 4 (13.8)	E6, 3 (10.3)
Phase 2‡					
2005, n = 369	CB5, 159 (43.1)	E18, 127 (34.4)	CB3, 47 (12.7)	E9, 25 (6.8)	CB1, 7 (1.9)
2006, n = 238	E25, 56 (23.5)	E30, 48 (20.2)	E5, 28 (11.8)	CA16, 20 (8.4)	CB4, 18 (7.6)
2007, n = 180	CB2, 62 (34.4)	CA9, 28 (15.6)	EV71, 21 (11.7)	E16, 14 (7.8)	CA10, 10 (5.6)
Phase 3§					
2008, n = 626	E30, 299 (47.8)	E6, 170 (27.2)	CA10, 33 (5.3)	CB3, 29 (4.6)	CB1, 27 (4.5)
2009, n = 288	EV71, 127 (44.1)	CB1, 70 (24.3)	CA2, 23 (8.0)	CA5, 20 (6.9)	CA14, 17 (5.9)
2010, n = 402	EV71, 190 (47.3)	CA6, 65 (16.2)	CB5, 32 (8.0)	CA9, 28 (7.0)	CA10, 19 (4.7)
2011, n = 529	EV71, 118 (22.3)	CA16, 109 (20.6)	CB5, 72 (13.6)	CB2, 70 (13.2)	E18, 42 (7.9)
Total, 1999–2011, n = 3,128	EV71, 476 (14.9)	E30, 390 (12.5)	CB5, 290 (9.3)	E6, 261 (8.4)	CB2, 186 (6.0)

*CB, coxsackievirus B; E, echovirus; EV, enterovirus; CA, coxsackievirus A.

**Figure 3:**
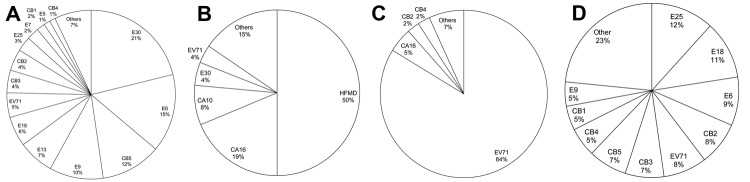
Distribution of nonpolio enterovirus genotypes by clinical manifestation. Graphics show percentage of each genotype from the total isolates of A) aseptic meningitis; B) hand, foot and mouth disease or herpangina; C), hand, foot and mouth disease with neurologic complications; and D), other pathogenesis including sepsis, acute gastroenteritis, hepatitis, pneumonia, and myopericarditis. CA, coxsackievirus A; CB, coxsackievirus B; E, echovirus; EV, enterovirus; HFMD, hand, foot and mouth disease.

EV genotypes are described in 4 major categories on the basis of associations with groups and clinical signs and symptoms; these are described as follows: 1) aseptic meningitis; 2) HFMD and/or herpangina; 3) HFMD with neurologic complications; and 4) other manifestations, including sepsis, acute gastroenteritis, hepatitis, pneumonia, and myopericarditis (Figure 3). The clinical manifestations of 1,624 (34.1%) patients whose samples tested positive were as follows (Figure 3): aseptic meningitis was diagnosed for 1,063 (65.5%) patients, HFMD for 155 (9.5%) patients, HFMD with neurologic complications for 295 (18.2%) patients, and other pathogenesis for 111(6.8%) patients. The genotypes of EV detected in 5 other cases during 1999–2011 are shown in in Figure 3. Aseptic meningitis was frequently associated with E30 (225/1,063, 21%), E6 (159/1,063, 15%), and CB5 (123/1,064, 12%) (Figure 3, panel A). Among HFMD cases, infection with CA16 was identified for 37% (58/155), CA10 for 16% (24/155), and E30 for 9% (14/155) of the patients (Figure 3, panel B). Regarding HFMD with neurologic complications, EV71 was the dominant genotype in 84% (247/295) of the cases and CA16 in 5% (14/295) (Figure 3, panel C). For patients with sepsis, acute gastroenteritis, hepatitis, pneumonia, and myopericarditis cases, E25, E18, and E6 were identified for 12% (13/111), 11% (12/111), and 9% (10/111), respectively (Figure 3, panel D).

## Discussion

We have presented longitudinal data reflecting changing patterns of enterovirus prevalence over a 13-year period in the South Korea while explicitly noting the changing laboratory methodology over the period. The results of prevalence and distribution of genotypes of EV in this study reflected nationwide outbreaks and detection and subtyping methods for EV surveillance.

We described 4 EV outbreaks in South Korea during 1999–2011: aseptic meningitis caused by EV71 in 2000, by E13 in 2002, by CB5 in 2005, and by E30 in 2008 (*25**–**27*). EV detection rates in 2002, 2005, and 2008 were relatively higher than those in other years, and the dominant genotypes found during these years were outbreak-associated genotypes.

The prevalence increased as detection technology changed from cell culture (phase I), to RT-PCR (phase II), to real-time RT-PCR (phase III). Overall increases were probably caused by a combination of outbreaks of EV infection and enhanced sensitivity of detection methods. Because of a greatly advanced molecular detection method, molecular-based methods enable detection of uncultivable EV by use of small sample quantities and specific primer sets. Consistent with our findings, Roth et al. reported higher sensitivity by using the RT-PCR method rather than cell culture for fecal and CSF samples (*11*). In our study, although there are no data from parallel testing to address the issue of relative sensitivity of the 3 detection methods used over this period in this study, it is possible that increased prevalence during phases II and III could have been accounted for by the enhanced sensitivity of detection methods.

We described the prevalence, seasonal trend, and epidemiologic data for human EV infection collected by the national enterovirus surveillance system during 1999–2011 in South Korea. Our laboratory identified EV from fecal, CSF, nasopharyngeal, blood, and other sample types such as urine from persons who had an array of symptoms. Although feces is the most convenient specimen type for detecting EVs for surveillance purposes, detecting EV in fecal samples is not the most specific way to confirm the cause of an individual patient's symptoms (*4*). In this study, a higher frequency of EV detection from fecal samples, in contrast to CSF, was observed; this finding is in agreement with the finding of a previous study that used both cell culture and RT-PCR (*11*). However, Antona et al. showed that when compared with other specimens, the highest percentage of positive detection was found in CSF specimens (*1*). This finding could be influenced by the fact that different detection methods were used for each sample type: Antona et al. used cell culture for fecal samples and RT-PCR for CSF.

Genotyping has been shown to greatly improve epidemiologic investigation of common EV types when compared with seroneutralization testing (*28*). During phase I of this study, CA types and some E and EV types did not propagate well in cell culture and, therefore, were underdiagnosed. Confirmation of EV genotype by sequencing was systematically conducted after 2005 in South Korea; untypeable EV decreased but reemerged as real-time RT-PCR methods were introduced for detection during 2008 (data not shown). These findings could be related to a low quantity of EV in the sample, which can be detected by using real-time PCR but not by PCR-based VP1 sequencing.

As far as clinical aspects are concerned, the prevalent ages and clinical manifestation are consistent with results from previous studies that showed that the majority of cases occurred in children <10 years of age (*1**,**29**,**30*). In addition, a link between clinical syndromes and genotypes was in accordance with previous studies (*1**, **2**,**4**–**6**,**31*); aseptic meningitis by E30, E6, and CB5; HFMD by CA16; and HFMD with neurologic complications by EV71. In this study, EV71 was the most frequent type of EV detected during 1999–2011 in South Korea. This finding is probably because our expanded surveillance detected more patients with neurologic disease. Since 1997, multiple cases of EV71 infection have been associated with severe aseptic meningitis and pulmonary edema in the Asia–Pacific region, including Taiwan, Malaysia, Singapore, and Japan (*26**,**32**–**37*). In addition, E30 was the second most common genotype detected during this period in this study and in previous studies from other countries; Asia and European countries reported that E30 was the predominant genotype (*1**,**4**,**11*).

EV infections have been known to increase in summer and early autumn in countries in temperate climates (*1**,**2**,**13*). As expected in a temperate climate, our surveillance data revealed a seasonal pattern of distribution, with transmission peaking in the summer and decreasing in the period from autumn to spring.

Our study has limitations that may affect the interpretation of its findings. First, although the patients were from almost all regions of South Korea, the number of patients and strains obtained from each is unequal. This variability is related to the level of cooperation and workload related to surveillance among different hospitals and local public health institutes. Second, some EV types that cannot propagate well in cell culture were underdiagnosed during 1999–2005, when ≈23.1% of isolates were recorded as untypeable (data not shown). Third, EV71 has been the most frequently detected type since 2009. It is likely that because HFMD with neurologic complications was actively monitored by our surveillance, EV serotypes associated with this clinical manifestation may have been overdetected.

This study focused on EV epidemiology in South Korea over a 13-year period by using a nationwide EV surveillance system. This surveillance provides valuable data on the epidemiologic pattern and clinical manifestations associated with specific genotypes and provides vital information that can be used to control annual EV epidemics. The public health impacts of EVs vary: some of the viruses are benign and some cause serious illness. Although it is appropriate in some instances to use cell cultures, we recommend the use of real-time RT-PCR for samples from patients who have typical symptoms of infection with the more virulent genotypes described here. Evaluation of findings from surveillance of enterovirus infections will contribute to development of prevention and treatment plans.
